# Hirschsprung's disease diagnosis: Comparison of immunohistochemical, hematoxilin and eosin staining

**DOI:** 10.4103/0971-9261.55153

**Published:** 2009

**Authors:** Mehrdad Memarzadeh, Ardeshir Talebi, Masod Edalaty, Mehrdad Hosseinpour, Nasrin Vahidi

**Affiliations:** Department of Surgery, Medical School, Isfahan University of Medical Sciences; 1 Department of Pathology, Medical School, Isfahan University of Medical Sciences and Department of Surgery, Medical School, Kashan University of Medical Sciences; 2 Al-Zahra University Hospital

**Keywords:** Hirschprung's Disease, immunohistochemical staining, megacolon

## Abstract

**Background:**

The diagnosis of Hirschsprung's disease (HD) is based on the absence of ganglion cells. In hemotoxilin and eosin (H and E) as well as acetylcholine esterase staining there are limitations in the diagnosis of immature ganglion cells in neonates.

**Methods:**

In this prospective study, 54 biopsies taken from suspected HD patients (five mucosal specimens and 49 full thickness specimens) were studied. In the laboratory, after preparing sections of paraffin embedded tissues, H and E staining slides were compared with immunohistochemical (IHC) staining including: S100, NSE, CD117, CD56, Cathepsin D, Vimentin, BCL2, GFAP, Synaptophysin and chromogranin.

**Results:**

The study revealed 30 negative (absence of ganglion cells) cases (55.5%), 17 positive cases (31.04%) and seven suspected cases (12.9%) of ganglion cells on the H and E staining. On IHC staining with CD56 and Cathepsin D, all of the 17 positive cases detected through H and E, were confirmed for having ganglion cells and out of 30 cases reported negative on H and E staining, 28(93.3%) were reported negative and two (6.7%) positive by IHC staining. Of the seven suspected cases H and E staining), IHC staining detectedganglion cells only in five slides; two remained negative.

**Conclusions:**

IHC staining using CD56 and Cathepsin D improved the accuracy of diagnosis in HD when used in addition to H and E staining technique, especially for negative or suspicious slides.

## INTRODUCTION

Hematoxilin and Eosin (H and E) staining, Acetylcholinesterase staining (AChE) are commonly used in the diagnosis of Hirschprung's disease (HD). However, diagnosis is not possible with H and E every times, because staining has limitations in the diagnosis of immature ganglion cells in neonates and the sub mucosal area in which the ganglion cells are small (three to five cells per ganglion) and irregularly distributed and so their identification is difficult and requires high expertise.[1,2]

On the other hand although AChE activity is diagnostically the most useful set of enzyme–histochemical reactions, it is not sufficient. AChE stains the parasympathetic nerve fibers and trunks of fibers that increase dramatically in the lamina propria mucosa and sub mucous layer, but is not a specific marker for ganglion cell.[3] AChE staining requires the experience of pathologists and in some instances interpretation is difficult.[4] There are reports of false positive and false negative results using this technique.[5]

Earlier, the importance of IHC studies has been emphasized in the diagnosis of immature ganglion cells, hypoganglionosis and other suspicious situations.[6–8] In this study, we compare IHC staining using neural markers with H and E staining to find out the best diagnostic panel for HD.

## MATERIAL AND METHODS

This is a prospective study conducted in the period 2001 to 2004. Rectal biopsy specimens from 54 infants suspected to be having HD constituted the material for the study. There were five mucosal and 49 full thickness biopsies. The specimens were kept in 10% formalin solution.

In the laboratory, after preparing sections of paraffin embedded tissues, H and E staining slides were compared with IHC staining including S_100_, NSE, CD_117,_ CD_56,_ Cathepsin D, Vimentin, BCL2, GFAP, Synaptophysin, chromogranin.

The slides for IHC were processed as follows:

- First sections of four *μ*m were obtained and fixed on the slides with polyelizine. This was followed by antigen retrieval for 10 minutes using heat and citrate buffer (pH is equal to six). Then 3% H_2_O_2_ and pure methanol were added for five minutes and sections were washed with distilled water. Next, primary antibody (with negative control) was added for 10 minutes and washing was performedSecondary antibody (biotinylated link) was added for 10 minutes and washed Streptavidin – HRP was added for 10 minutes and washed with P.B.S. After adding substrate chromogen (D.A.B) for 10 minutes, counter was stained. All the antibodies were from DAKO.- Co and the slides were scanned for ganglion cells.

The best staining method was appreciated based on the degree of staining of ganglion cell versus its background and clear detection of ganglion cells.

## RESULTS

**Figure 1:**
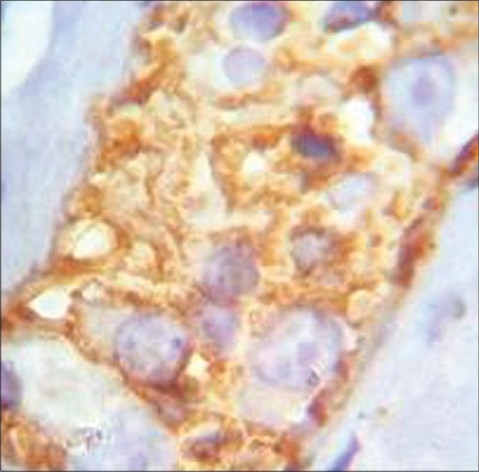
IHC staining of CD56. In Catepsin D staining, the ganglion cells are stainable; but the background is not. (Magnification =10×40)

**Figure 2:**
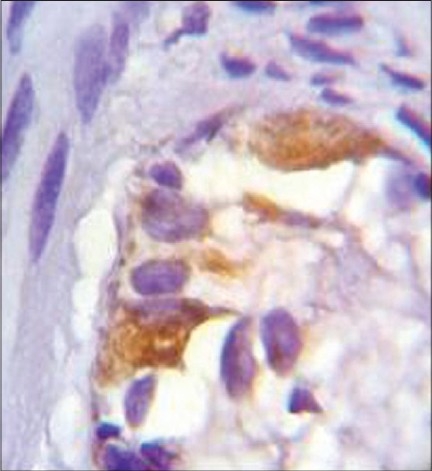
IHC staining of Cathepsin D. In CD56 staining the background is stainable; but ganglion cells are not. (Magnification =10×40)

In the microscopic study of 54 specimens, H and E staining revealed absence of ganglion cells (negative) in 30 cases (55.5%), presence of cells (positive) in 17(31.04%) and suspected presence in seven cases (12.9%). In the study of specimens through IHC staining with CD56 and Cathepsin D [Figure 1 and 2], all 17 cases detected positive through H and E, were confirmed for having ganglion cells and of the 30 cases reported negative through H and E staining, 28 (93.3%) were reportednegative and two (6.7%) positive by IHC. Out of seven cases suspected of having ganglion cells on H and E staining, we could find ganglion cells in five slides while two remained negative [Table 1].

**Table 1:** Comparison of Detection of Ganglion Cells in H& E and IHC staining

	H and E Positive (17)	H and E Negative (30)	H and E Suspicious (7)
IHC Positive	17	2	5
IHC Negative	0	28	2

According to our selection criteria, other markers (S100, NSE, CD_117,_ Vimentin, bcl2, GFAP, Synaptophysin, and chromogranin)[9] used for IHC staining were suboptimal in comparison to CD56 and Cathepsin D [Figure 3].

**Figure 3A:**
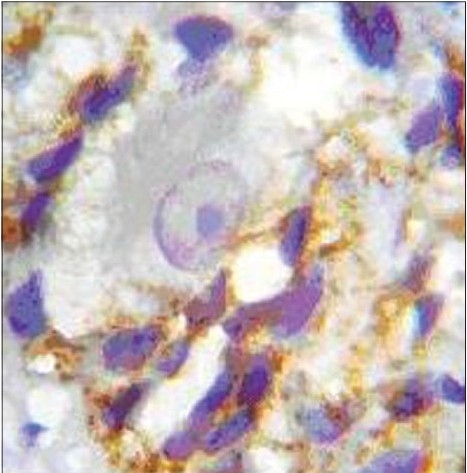
IHC staining of (A) Synaptophysin and (B) BCL2. (In Synaptophysin staining, the ganglion cells and background are not stainable well, but in BCL2 staining, the ganglion cells and background are both stainable. (Magnification 10 × 40)

**Figure 3B:**
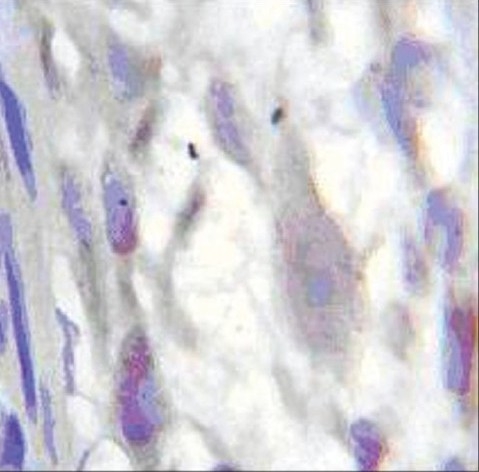
IHC staining of (A) Synaptophysin and (B) BCL2. (In Synaptophysin staining, the ganglion cells and background are not stainable well, but in BCL2 staining, the ganglion cells and background are both stainable. (Magnifications =10 × 40)

## DISCUSSION

Detection of ganglion cells in H and E sections can be a difficult process for the pathologist.[10] The maturation of ganglion cells is incomplete at the time of birth, especially in the sub mucosal area.[7] Immature ganglion cells may be unipolar or bipolar and can be mistaken for stromal cells.[7] Sub mucosal ganglion cells are smaller than myenteric plexus ganglion cells,[2] and pathologists have to prepare between 50 to 400 sections of H and E stained slides to find ganglion cells.[11] On the other hand, although AChE staining is the chosen technique for some pathologists[5] its diagnosis needs experience and its interpretation is difficult in some instances.[4] One of the problems is the interference of red blood cell (RBC) acetyl cholinesterase due to hemorrhage in lamina propria.[10] Also, false positive[11] and false negative[10] reactions were reported using this staining technique. Technical difficulties and storage problem of reagents is also reported.[5,12,13,16–18]

Park *et al*.[10] found that the main diagnostic pitfall was the interpretation of the enteric nervous plexuses in the transitional zone and the detection of the indistinct or immature neurons indistinguishable from enteric glial cells or satellite cells. They showed immunohistochemical study was a very helpful diagnostic adjunct to delineating the immature neurons (BCL2), and the size of the enteric ganglia and neuromuscular innervation (S-100 protein, Synaptophysin, and CD56). Another study[12–14] found that Synaptophysin-positive synapses distribution in circular and longitudinal colonic muscles and intermuscular ganglions can reflect functional disturbances of large bowel motility and could be helpful in the description of the innervation status of colonic specimens in HD patients.

Facing a wide diversity of opinions, we decided to compare IHC markers with H and E staining to find out the best diagnostic panel for detection of ganglion cells. As shown in figures 1 to 3, ganglion cell detection and its staining with CD56 and Cathepsin D were better than BCL2 and Synaptophysin.

To conclude, our study shows that IHC markers, including both Cathepsin D and CD56, especially for negative or suspicious slides are the best diagnostic panel for detection of ganglion cells. In Cathepsin D staining, the ganglion cells are stainable, but the background is not. On the contrary, in CD56 staining the background is stainable, but ganglion cells are not, these two methods complement each other. This panel can detect smaller or immature ganglion cells and also small cytoplasmic portions of those cells. Hence, the sensitivity and specificity are increased and false negative and positive results are decreased.
